# Paranoia and belief updating during a crisis

**DOI:** 10.21203/rs.3.rs-145987/v1

**Published:** 2021-01-18

**Authors:** Praveen Suthaharan, Erin J. Reed, Pantelis Leptourgos, Joshua Kenney, Stefan Uddenberg, Christoph D. Mathys, Leib Litman, Jonathan Robinson, Aaron J. Moss, Jane R. Taylor, Stephanie M. Groman, Philip R. Corlett

**Affiliations:** 1 Department of Psychiatry, Connecticut Mental Health Center, Yale University, New Haven, CT, USA; 2 Interdepartmental Neuroscience Program, Yale School of Medicine, New Haven, CT, USA.; 3 Yale MD-PhD Program, Yale School of Medicine, New Haven, CT, USA.; 4 Princeton Neuroscience Institute, Princeton University, Princeton, NJ, USA.; 5 Interacting Minds Center, Aarhus University, Aarhus, Denmark; 6 Translational Neuromodeling Unit (TNU), Institute for Biomedical Engineering, University of Zurich and ETH Zurich, Zurich, Switzerland; 7 CloudResearch, 65-30 Kissena Blvd Hall 2, Room 20, Flushing, NY 11367

## Abstract

The 2019 coronavirus (COVID-19) pandemic has made the world seem unpredictable. During such crises we can experience concerns that others might be against us, culminating perhaps in paranoid conspiracy theories. Here, we investigate paranoia and belief updating in an online sample (N=1,010) in the United States of America (U.S.A). We demonstrate the pandemic increased individuals’ self-rated paranoia and rendered their task-based belief updating more erratic. Local lockdown and reopening policies, as well as culture more broadly, markedly influenced participants’ belief-updating: an early and sustained lockdown rendered people’s belief updating less capricious. Masks are clearly an effective public health measure against COVID-19. However, state-mandated mask wearing increased paranoia and induced more erratic behaviour. Remarkably, this was most evident in those states where adherence to mask wearing rules was poor but where rule following is typically more common. This paranoia may explain the lack of compliance with this simple and effective countermeasure. Computational analyses of participant behaviour suggested that people with higher paranoia expected the task to be more unstable, but at the same time predicted more rewards. In a follow-up study we found people who were more paranoid endorsed conspiracies about mask-wearing and potential vaccines – again, mask attitude and conspiratorial beliefs were associated with erratic task behaviour and changed priors. Future public health responses to the pandemic might leverage these observations, mollifying paranoia and increasing adherence by tempering people’s expectations of other’s behaviour, and the environment more broadly, and reinforcing compliance.

## Introduction

Crises, from terrorist attacks^1^ to viral pandemics, are fertile grounds for paranoia^2^, the belief that others bear malicious intent towards us. Paranoia may be driven by altered social inferences^3^, or by domain-general mechanisms for processing uncertainty^4, 5^. The COVID-19 pandemic increased real-world uncertainty and provided an unprecedented opportunity to track the impact of an unfolding crisis on human beliefs.

**Figure 1.**
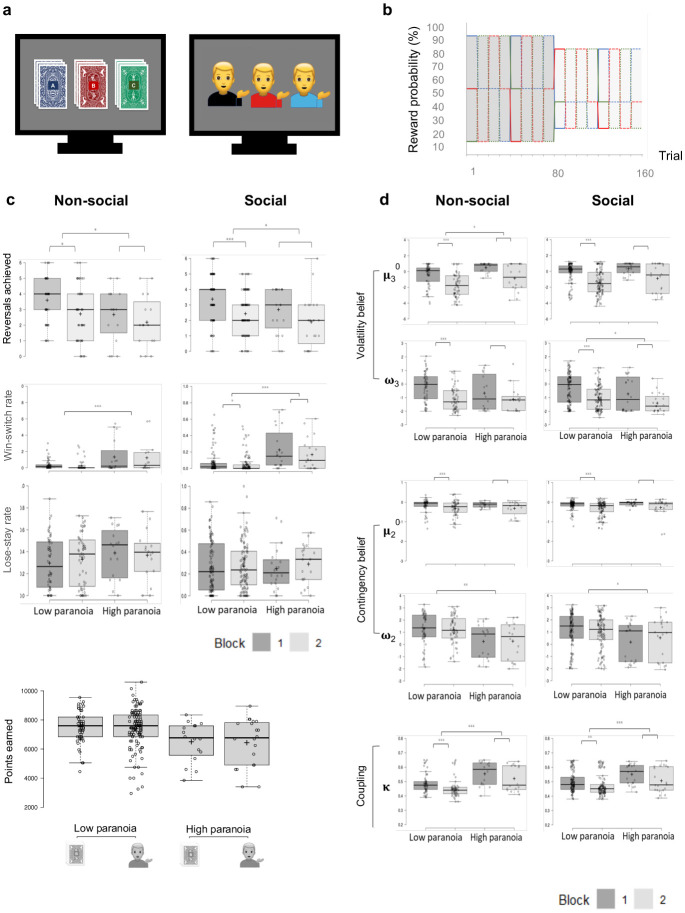
Pre-pandemic social and non-social reversal learning. **a,** non-social and social task stimuli. **b,** reward contingency schedule. **c,** in both non-social and social tasks, paranoid subjects achieve fewer reversals, switch more frequently after positive feedback (”win-switch rate”). **d,** High paranoia subjects exhibit elevated priors for volatility and contingency beliefs (**μ**_2_^0^ and **μ**_3_^0^), are slower to update those beliefs (**ω**_2_, **ω**_3_), and have higher coupling between volatility and contingency beliefs (***κ***)**. Box-plots:** Centre lines show the medians; box limits indicate the 25th and 75th percentiles; whiskers extend 1.5 times the interquartile range from the 25th and 75th percentiles, outliers are represented by dots; crosses represent sample means; data points are plotted as open circles. *P ≤ 0.05, **P ≤ 0.01, ***P ≤ 0.001.

We examined self-rated paranoia^6^ alongside social and non-social belief updating in computer-based tasks (Figure 1a), spanning three time periods: before the pandemic lockdown, during lockdown, and into reopening. We further explored the impact of state-level pandemic responses on beliefs and behaviour. We hypothesized that paranoia would increase during the pandemic, perhaps driven by the need to explain and understand real-world volatility^1^. Furthermore, we expected that real-world volatility would change individuals’ sensitivity to task-based volatility, causing them to update their beliefs in a computerized task accordingly^5^ . Finally, since different states responded more or less vigorously to the pandemic, and the residents of those states complied with those policies differently, we expected that efforts to quell the pandemic would change perceived real-world volatility, and thus paranoid ideation and task-based belief updating.

**Figure 2.**
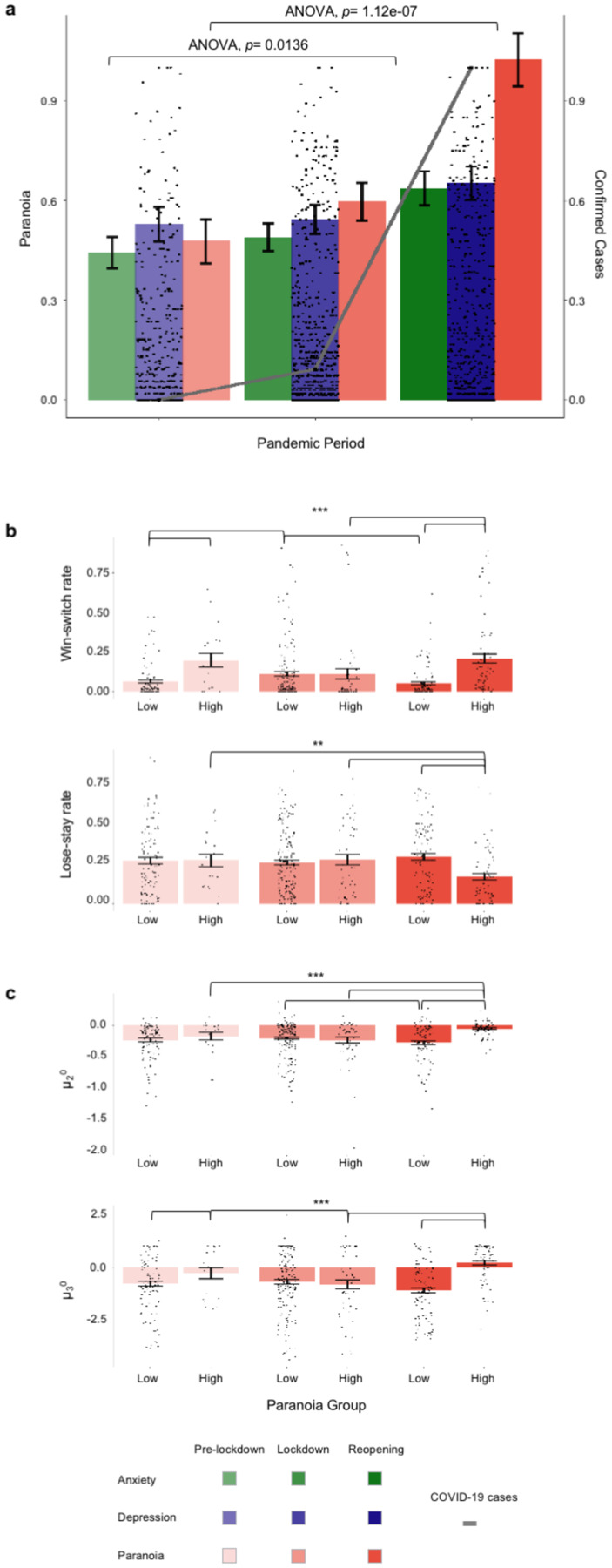
Paranoia, depression, anxiety, task behaviour, and belief updating during a pandemic. Paranoia increased as the pandemic progressed. **a,** self-rated paranoia, depression, and anxiety alongside normalized confirmed cases of COVID-19, prior to the pandemic, during lockdown and following reopening. **b,** win-switch and lose-stay behaviours in reversal learning task for low versus high paranoia participants prior to the pandemic, during lockdown and following reopening. **c,** Expected reinforcement (***μ***_2_^0^) and volatility (***μ***_3_^0^) in task, estimated by model inversion for high and low paranoia participants. *P ≤ 0.05, **P ≤ 0.01, ***P ≤ 0.001.

The pandemic significantly increased self-rated paranoia from January 2020 through the lockdown, peaking during reopening (F_(2, 530)_=16.5, p= 1.12E-7, η_p_^2^=1.00), mirroring the increase in confirmed COVID-19 cases (Figure 2a). However, depression (F_(2, 530)_=1.87, p= 0.156, η_p_^2^=1.00) did not increase significantly. Anxiety increased (F_(2, 530)_=4.34, p= 0.014, η_p_^2^=1.00) but, the change was less pronounced than paranoia (Figure 2a), suggesting a particular impact of the pandemic on beliefs about others.

### Relating paranoia to task-derived social and non-social belief updating

We administered a probabilistic reversal learning task. Participants chose between options with different reward probabilities to learn the best option (Figure 1b)^7^. They were forewarned that the best option may change, but not when or how often^7^. Hence, the task assayed belief formation and updating under uncertainty^7^. The challenge is to harbour beliefs that are robust to noise but sensitive to real changes in reward contingencies^7^.

Before the pandemic, people who were more paranoid (scoring in the clinical range on standard scales^6, 8^) were more likely to switch their choices between options, even following positive feedback^5^. We compared those data (gathered via the *Amazon Mechanical Turk Marketplace* in the U.S.A. between December 2017 and August 2018) to a new task version with identical contingencies, but framed socially (Figure 1a). Instead of selecting between decks of cards (‘non-social task’), participants chose between three potential collaborators who might increase or decrease their score. These data were gathered during January 2020, before the World Health Organization declared a global pandemic. Participants with higher paranoia switched more frequently than low paranoia participants after receiving positive feedback in both the social and non-social tasks (Figure 1c; win-switch rate: social task, F_(1, 128)_=19.855, p=1.80E-5, η_p_^2^=0.134; non-social task, F_(1, 70)_=12.698, p=0.001, η_p_^2^=0.154). High and low paranoia participants did not differ in their perseveration after negative feedback (lose-stay rate: social task, F_(1, 128)_=0.004, p=0.948, η_p_^2^=0.000034; non-social task, F_(1, 70)_=1.095, p=0.299, η_p_^2^=0.015). There were no significant differences in the impact of paranoia on social and non-social reversal learning behaviors.

### Computational modelling

In order to dissect the mechanisms of belief updating, we aligned participants’ choices with a computational model and estimated its parameters^9, 10^, comparing their magnitudes between groups and tasks^11^, before and after the pandemic.

Our generative model, the hierarchical Gaussian filter^9, 10^, is comprised of three hierarchical layers of belief about the task, represented as probability distributions which encode belief content and uncertainty: (1) reward belief (what was the outcome?), (2) contingency beliefs (what are the current values of the options [decks/collaborators]?), and, (3) volatility beliefs (how do option values change over time?). Each layer updates the layer above it in light of evolving experiences, which engender prediction errors and drive learning proportionally to current variance. Each has an initial mean ***μ***^**0**^, a prior belief. ***ω*** encodes the impact of tonic uncertainty on belief updating. ***κ*** captures sensitivity to perceived phasic changes in the task. These beliefs are summed and fed through a sigmoid response function whose temperature is inversely proportional to the estimated task volatility (thus decisions are more stochastic under higher volatility). Using this model we have previously demonstrated identical belief updating deficits in paranoid humans and rats administered methamphetamine^5^, and that this model better captures participants’ responses to volatility and the effects of paranoia on those responses, compared to standard reinforcement-learning models^5^

Before the pandemic, high paranoia participants exhibited elevated ***κ*** – they were overly sensitive to perceived abrupt changes in the reinforcement probabilities (social task, F_(1, 128)_=7.773, p=0.006, η_p_^2^=0.057; non-social task, F_(1, 70)_=13.644, p=0.0004, η_p_^2^=0.163; MD_META_=0.053, CI_META_=[0.027, 0.078], z_META_=4.035, p_META_=5.45E-5). However, ***ω***_2_ was lower in high paranoia, indicating that tonic task changes were less impactful on their choices (Fig. 1a; social task, F_(1, 128)_=5.091, p=0.026, η_p_^2^=0.038; non-social task, F_(1, 70)_=8.681, p=0.004, η_p_^2^=0.11). Across social and non-social contexts, high paranoia participants expected more volatility (***μ***_3_^0^, MD_META_=0.6749, CI_META_=[0.2527, 1.0971], z_META_=3.1332, p_META_=0.0017) and were slower to adjust this belief than controls (***ω***_3,_ MD_META_= −0.3361, CI_META_=[−0.6342, −0.0380], z_META_=−2.2099, p_META_=0.0271), favoring a domain-general account of paranoia (Figure 1d)^4^.

### The impact of an evolving pandemic on paranoia and belief updating

After the pandemic was declared we continued to acquire data on both tasks (3/19/2020–7/17/2020). We found an interaction between paranoia and pandemic period for win-switching (F_(2, 593)_=9.075, p=0.0001, η_p_^2^=0.030, Figure 2b). High paranoia participants win-switched more than low paranoia participants before the lockdown (MD_EMM_=0.116, SE_EMM_= 0.031, p_EMM_=0.0002) and during reopening (MD_EMM_=0.153, SE_EMM_= 0.026, p_EMM_=5.87E-9). High and low paranoia did not differ in their win switching during lockdown (MD_EMM_<0.001, SE_EMM_= 0.027, p_EMM_=0.987). Again, consistent with a domain-general account^4^, there were no differences between behaviour in the social and non-social tasks. In sum, reopening increased irrational win-switching in more paranoid participants.

Volatility priors (***μ***_3_^0^) and coupling (***κ***) both exhibited interactions between pandemic period and paranoia (***μ***_3_^0^: F_(2, 593)_=4.811, p=0.009, η_p_^2^=0.016; ***κ***: F_(2, 593)_=5.766, p=0.003, η_p_^2^=0.019). Volatility priors and coupling were higher in paranoid participants before pandemic lockdown (***μ***_3_^0^: p_EMM_=0.002, ***κ***: p_EMM_=1.67E-5) and during reopening (***μ***_3_^0^: p_EMM_=4.42E-7, ***κ***: p_EMM_=0.002). During lockdown, the paranoia groups did not differ (***μ***_3_^0^, p_EMM_=0.314). During reopening ***μ***_3_^0^ increased only in high paranoia subjects (MD_EMM_=0.837, SE_EMM_=0.218, p_EMM_=0.0001). It appears that lockdown had a mollifying effect in high paranoia, perhaps by enforcing avoidance behaviours^12^, decreasing social interaction and thus assuaging concerns about others (Figure 2c).

Lose-stay rates also exhibited a period by paranoia interaction (F_(2, 593)_=6.51, p=0.002, η_p_^2^=0.021, Figure 2b). During reopening, high paranoia participants were less likely than participants with low paranoia to persist after negative feedback. Lose-stay rates declined in high paranoia participants on reopening. In parallel, we observed an increase in their contingency prior (***μ***_2_^0^) after reopening (F_(2, 593)_=8.996, p=0.0001, η_p_^2^=0.029, Figure 2c). Across the three pandemic periods, ***μ***_2_^0^ correlated negatively with lose-stay behavior (r=−0.69, p=1.3E-7). These findings suggest that paranoid subjects had higher expectations of reward during reopening and were less likely to tolerate negative feedback.

Specifically, low paranoia appeared to temper reward expectations. Tonic belief updating parameters showed a paranoia group effect (***ω***_3_: F_(1, 593)_=19.31, p=1.32E-5, η_p_^2^=0.032), and a significant block by paranoia interaction (***ω***_2_: F_(1, 593)_=5.446, p=0.02, η_p_^2^=0.009). High paranoia subjects were slower to update their volatility and reinforcement beliefs.

We asked participants in the social task to rate whether or not they believed that the avatars had deliberately sabotaged them. Win-switch rate (r=0.259, p=1.2E-5, n=280), ***μ***_2_^0^ (r=0.124, p=0.038), and ***μ***_3_^0^ (r=0.154, p=0.01) – parameters that are elevated in paranoid participants – were positively correlated with sabotage belief. These findings suggest that participants with higher paranoia expected more positive interactions with the avatars initially. Those expectations were quickly confounded, garnering beliefs that the avatars had nefarious intentions.

### Effects of the pandemic on paranoia and task behaviour

Within the U.S.A., states responded differently to the pandemic; some instituted lockdowns early and broadly, whereas others closed later and reopened sooner. When they reopened, some states mandated mask wearing. Others did not.

**Figure 3.**
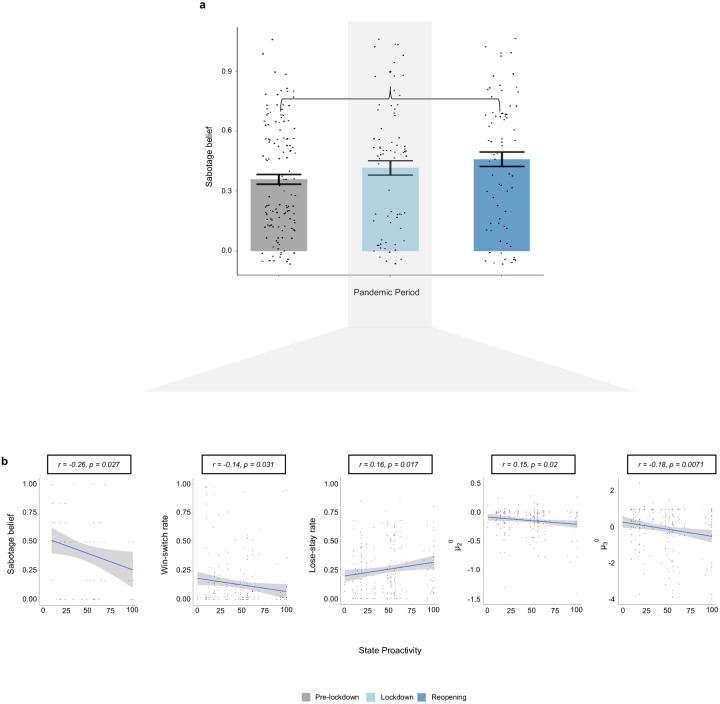
Sabotage belief and the effects of lockdown (social task). **a,** sabotage belief, the conviction that an avatar-partner deliberately caused a loss in points, increased as the pandemic progressed through pre-pandemic, lockdown, and reopening periods **b,** State proactivity in lockdown (earlier intervention with prolonged duration) correlated with decreased sabotage belief, decreased win-switch rate, increased lose-stay rate, lower expected reinforcement and lower expected volatility.

The win-switch data, ***κ***, and ***μ***_3_^0^ estimates suggest that lockdown ameliorated learning disturbances in paranoid subjects. Whereas sabotage belief generally increased with pandemic period (m_pre-lockdown_ = 0.36, m_reopening_ = 0.46, t_(145)_, *p* = 0.02, Figure 3a), proactive state lockdown responses (earlier lockdown, later reopening) correlated negatively with sabotage belief (r=−0.26, p=0.027, Fig 3b). These data suggest that early and decisive state interventions may have mitigated paranoia during the escalating uncertainty of lockdown.

### Is paranoia induced by mask-wearing policies?

We recorded a significant increase in paranoia when Americans were emerging from lockdown (Figure 2A). We wondered what might be contributing to that effect. Mask wearing in public became more common and necessary at that time. Some states imposed a mask wearing mandate and others did not. Following a quasi-experimental approach to causal inferences (developed in econometrics and recently extended to behavioural and cognitive neuroscience^13^), we pursued a difference-in-differences (DiD) analysis to discern the effects of state mask-wearing policy on paranoia. A DiD design compares changes in outcomes before and after a given policy takes effect in one area, to changes in the same outcomes in another area that did not introduce the policy^14^. The data must be longitudinal, but they needn’t follow the same participants^14^. Before pursuing such an analysis, it is important to establish parity between the two comparator locations^15^, so that any differences can be more clearly ascribed to the policy that was implemented. We believe such parity obtains in our case. First, there were no significant differences at baseline (in May) in the number of cases or deaths in states that went on to mandate versus recommend mask wearing (cases, t=−2.02, d.f.=8.24, p=0.07, deaths, t=−1.68, d.f.=8.19, p=0.13). Furthermore, paranoia is held to flourish during periods of economic inequality^16^. There were no baseline differences in unemployment rates in May (prior to the mask policy onset) between states that mandated masks versus states that recommended mask wearing (t=−1.07, d.f.=11.6, p=0.31). We employed a between participants design, so it is important to establish that there were no demographic differences (age, gender, race) in participants from states that mandated versus participants from states that recommended mask-wearing (age, t=−1.46, d.f. = 42.5, p=0.15, gender, χ^2^=0.37, d.f.=1, p=0.54, race, Fisher’s exact test for count data, p=0.21). On these bases, we chose to proceed with the DiD analysis.

**Figure 4.**
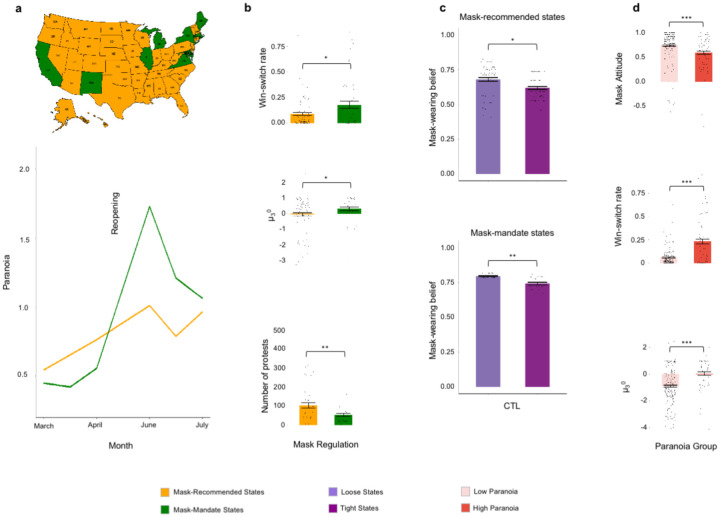
Effects of mask policy on paranoia and belief-updating. We observe a significant increase in paranoia and perceived volatility, especially in states that have issued a state-wide mask mandate. **a**, Map of the US states color-coded to their respective mask policy and a Differences-in-Differences analysis (bottom) of mask rules suggests a 48% increase in paranoia in states that mandate mask-wearing. **b**, Win-switch rate (top) and volatility belief (middle) are higher in mask-mandate states, and more protests per day in mask-recommended states (bottom). **c**, Effects of Cultural Tightness and Looseness (CTL) in mask-recommended states (top) and mask-mandate states (bottom) implicating violation of social norms in the genesis of paranoia. **d**, Follow-up study illustrating that high paranoia participants are less inclined to wear masks in public (top), have more promiscuous switching behaviour (middle) and elevated prior beliefs about volatility (bottom).

Mandated mask wearing was associated with an estimated 48% increase in paranoia (γ_DID_ = 0.48, *p* = 0.018), relative to states in which mask wearing was recommended but not required (Figure 4a). This increase in paranoia was mirrored as significantly higher win-switch rates in participant task performance (two-sample: m_rec_ = 0.09, m_req_ = 0.18, t_67_ = −2.4, *p* = 0.02) as well as stronger volatility priors (***μ***_3_^0^, marshalling data from both tasks, two-sample: m_rec_ = −0.06, m_req_ = 0.30, t_125_ = −2.1, *p* = 0.036 Figure 4b).

### Does variation in rule following contribute to the increase in paranoia?

We examined whether any other features might illuminate this variation in paranoia by local mask policy^17^. There are state-level cultural differences – measured by the Cultural Tightness and Looseness (CTL) index^17^ – with regards to rule following and tolerance for deviance. Tighter states have more rules and tolerate less deviance, whereas looser states have few strongly enforced rules and greater tolerance for deviance^17^. We also tried to assess whether people were following the mask rules. We acquired independent survey data gathered in the U.S.A. from 250,000 respondents who, between July 2 and July 14, were asked: *How often do you wear a mask in public when you expect to be within six feet of another person?*^18^ These data were used to compute an estimated frequency of mask wearing in each state during the reopening period (Figure 4c).

We found that in culturally tighter states where mask wearing was mandated, mask wearing was lowest (m_loose_=0.787, m_tight_=0.760, t_32_=2.87, p=0.007). Furthermore, even in states where mask wearing was recommended, mask wearing was lowest in culturally tighter states (m_loose_=0.674, m_tight_=0.629, t_107_=2.46, p=0.016).

**Figure 5.**
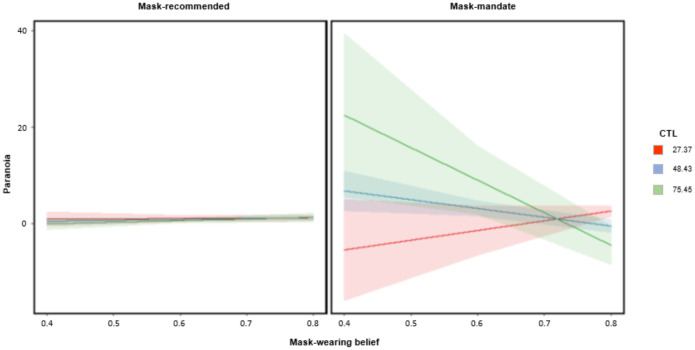
Predicting paranoia from pandemic features. Regression model predictions in states where masks were recommended (Left Panel) versus mandated (right panel). Paranoia predictions based on estimated state mask-wearing (x-axis, low mask-wearing to high mask-wearing) and cultural tightness. **Red** – Loose states, that do not prize conformity, **Blue -** states with median tightness, **Green** – tight states that are conservative and rule-following. Paranoia is highest when mask wearing is low, in culturally tight states with a mask-wearing mandate.

Through backward linear regression with removal, we fit a series of models attempting to predict individuals’ self-rated paranoia (N=172) from the features of their environment, including whether they were subject to a mask mandate or not, the cultural tightness of their state, state-level mask-wearing, and Coronavirus cases in their state. In the best fitting model (F_(11,160)_=1.91,p=0.04) there was a significant three way interaction between mandate, state tightness and perceived mask wearing (t_24_=−2.4, p=0.018) – paranoia was highest in mandate state participants living in areas that were culturally tighter, where fewer people were wearing masks (Figure 5). Our analyses imply that mask-wearing mandates and their violation, particularly in places that value rule following, may have increased paranoia. Alternatively, the mandate may have increased paranoia in culturally conservative states, culminating in less mask wearing.

### How is paranoia related to beliefs about mask-wearing?

In a follow-up study, we attempted a conceptual replication, recruiting a further 405 participants (between 09/06/20 and 11/02/20), polling their paranoia, their attitudes toward mask-wearing, and capturing their belief updating under uncertainty with the probabilistic reversal learning task. Individuals with high paranoia were more reluctant to wear masks and reported wearing them significantly less (t_157_ = −4.3, p = 2.45E-05). Again, win-switch rate was significantly higher in high paranoia individuals (t_99_ = 6.4, p = 5.08E-09), as was their prior belief about volatility (t_157_ = 6.4, p = 1.60E-09), confirming the links between paranoia, mask hesitancy, erratic task behaviour and expected volatility that our DiD analysis suggested (Figure 4d). Our data imply that paranoia flourishes when individuals’ attitudes conflict with what they are being instructed to do, particularly in areas where rule following is more common – paranoia may be driven by a fear of social reprisals for one’s anti-mask attitudes.

### Other changes that were coincident with the onset of mask policies

In addition to the pandemic, other events have increased unrest and uncertainty, notably the protests following the killings of George Floyd and Breonna Taylor. These protests began on May 24^th^ 2020 and continue, occurring in every US state. To explore the possibility that these events were contributing to our results, we compared the number of protest events in mandate and recommended states in the months before and after reopening. There were significantly more protests per day from May 24th through July 31^st^ 2020 in mask-recommended states versus mask-mandated states (t_87_=3.10, p=0.0027). This suggests the effect of mask mandates we observed was not driven by the coincidence of protests and reopening, indeed, protests were less frequent in states with higher paranoia (Figure 4b).

Whilst mask-mandate and mask-recommend states were matched at baseline, it is possible that increases in cases and deaths at reopening explain the increase in paranoia, rather than the mask mandate. Our data militate against this explanation.

There were no significant differences in cases (t=−1.79, d.f.=8.95, p=0.11) or deaths (t=−1.82, d.f.=8.30, p=0.10) during reopening in mandate versus recommend states. Furthermore, self-rated contamination fears^19^ actually significantly decreased at reopening relative to lockdown (t=2.73, d.f.=356.47, p=0.0067), when paranoia peaked, and were significantly higher in mask-recommended states compared to mask mandate states (t=2.77, d.f.=109.85, p=0.0066). Thus, cases, deaths, and concerns about being contaminated did not track the increase in paranoia we observed in mandate states. These data are consistent with the increase in paranoia being centred on the onset of the mask mandate, rather than other features that may have been coincident with reopening.

### Did changes in the online participant pool drive the effects?

**Figure 6.**
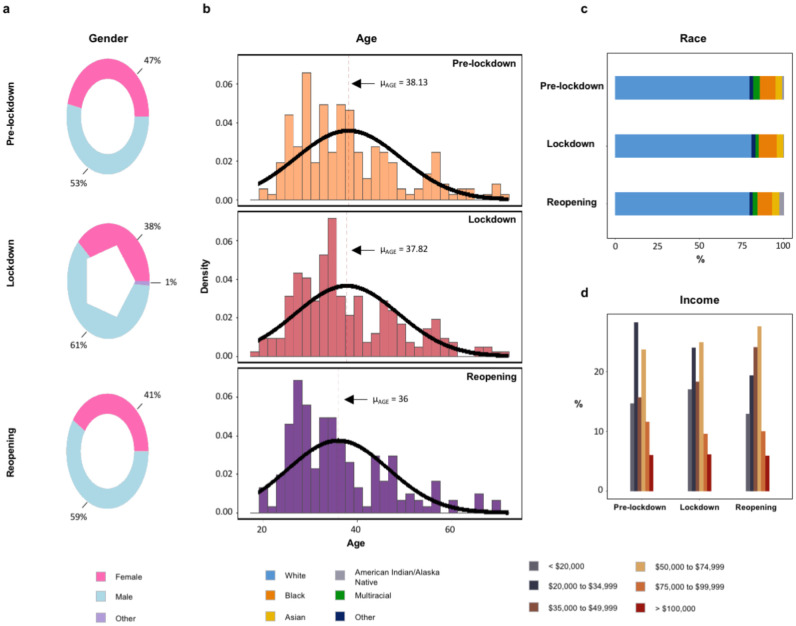
Demographics across the pandemic periods. **a)** Gender, **b)** Age, **c)** Race and **d)** Income compositions for each period. We demonstrate consistent demographic distributions from pre-lockdown into reopening

**Figure 7.**
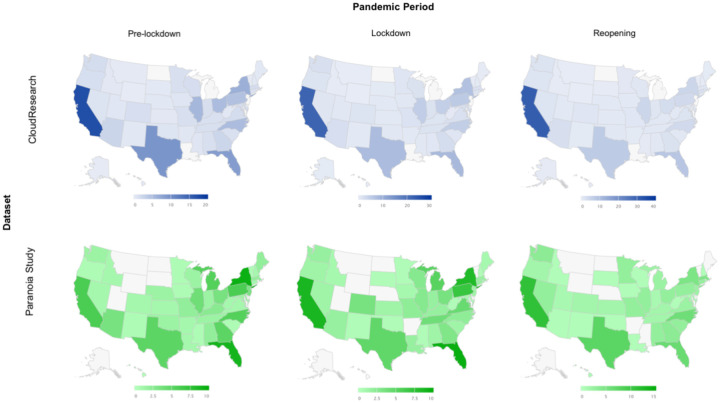
Geographic comparison of our paranoia study (Green) to CloudResearch’s data (Blue). We compare the sampling of US CloudResearch participants between the large CloudResearch data platform and our pandemic dataset. The blue maps represent mean percentage of participant recruitment per state across CloudResearch-hosted studies for each period (*pre-lockdown*: N= 6648 studies; *lockdown*: N= 177 studies; *reopening*: N= 468 studies). The green maps represent mean percentage of participant recruitment per state in our pandemic study alone for each period.

Given that the pandemic has altered our behaviour and beliefs, it is critical to establish that the effects we describe above are not driven by changes in sampling. For example, with lockdown and unemployment, more people may have been available to participate in online studies. We find no differences in demographic variables (age F_2,392_=1.991, p=0.14, gender χ^2^=2.81 d.f.=2, p=0.25, race χ^2^=7.61, d.f.=10, p=0.67, income, χ^2^=8.68, d.f.=10, p=0.56) across our study periods (pre-pandemic, lockdown, reopening, Figure 5). Furthermore, given that the effects we describe depend on geographical location, we confirm that the proportions of participants recruited from each state did not differ across our study periods (χ^2^=6.63, d.f.=6, p=0.34, Figure 6). Finally, in order to assuage concerns that the participant pool changed as the result of the pandemic, published analyses confirm that it did not^20^. Furthermore, in collaboration with CloudResearch^21^, we ascertained location data spanning our study periods from 7,293 experiments comprising 2.5 million participants. The distributions of participants across states match those that we recruited, and the mean proportion of participants in a state across all studies in the pool for each period correlates significantly with the proportion of participants in each state in the data we acquired for each period: pre-pandemic, r = 0.76 p = 2.2E-8; lockdown, r = 0.78 p = 5.8E-9; reopening, r = 0.81 p = 8.5E-10 (Figure 6). Thus, we did not, by chance, recruit more participants from mask-mandating states or tighter states, for example. Furthermore, focusing on the data that went into the DiD, there were no demographic differences pre- versus post-reopening for mask-mandate versus mask-recommended states (age, p=0.45, gender, p=0.73, race, p=0.17, Figure 7). Taken together with our task and self-report results, these control analyses increase our confidence that during reopening, people were most paranoid in the presence of rules and perceived rule breaking, particularly in states where people usually tend to follow the rules.

### Paranoia versus conspiracy theorizing

**Figure 8.**
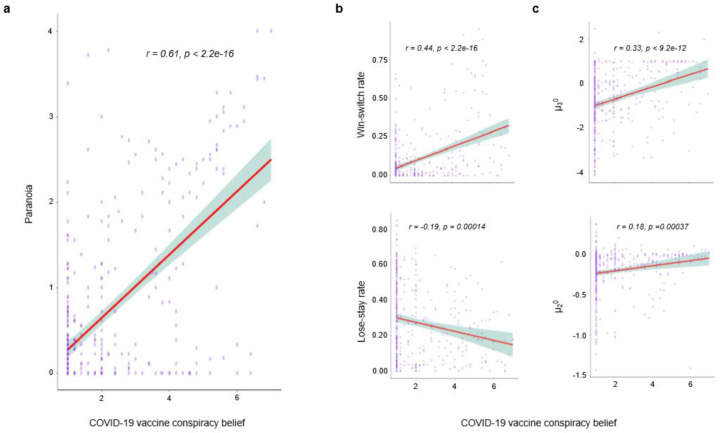
Relating vaccine conspiracy beliefs to paranoia and task behaviour. We assayed individual’s COVID-19 vaccine conspiracy beliefs to investigate underlying relationships to behaviour. We find individuals with higher paranoia endorsed more vaccine conspiracies relative to their lower paranoia counterparts. Similarly, beliefs were strongly correlated with erratic task behavior – increased win-switching and decreased lose-stay – and perturbed priors.

Whilst correlated, paranoia and conspiracy beliefs are not synonymous^22^. Therefore, we also assessed conspiracy beliefs about a potential COVID vaccine. We found that conspiracy beliefs about a vaccine correlated significantly with paranoia (r= 0.61, p < 2.2E-16), and that such beliefs were associated with erratic task behaviour (win-switch rate: r=0.44, p < 2.2E-16; lose-stay rate: r=−0.19, p=0.00014) and perturbed priors (***μ***_3_^0^: r=0.33, p < 9.2E-12; ***μ***_2_^0^: r=0.18, p = 0.000037) in an identical manner to mask concerns and paranoia more broadly (Figure 8).

## Discussion

The COVID-19 pandemic increased paranoia in a manner that correlated with the number of confirmed cases. During reopening, wherein paranoia peaked, win-switch behaviour likewise increased significantly in high paranoia participants across both social and non-social tasks. Paranoia appears related to domain-general rather than selectively social inference processes^5^. Regardless of local policies, paranoid subjects were slower to update volatility priors and showed elevated coupling between volatility and contingency beliefs. ***μ***_3_^0^ correlated with stronger beliefs in the nefarious intentions of others in the social task.

The lockdown rendered participants in less proactive states more susceptible to paranoia in terms of their expectations about volatility. However, we also found that people who were less paranoid during lockdown and reopening were more forgiving of collaborators, returning to those characters even after they have delivered losses in the social task.

The increase in paranoia that we observed appeared to coincide with reopening from lockdown and to be particularly pronounced in states that mandated that their residents wear masks when in public. We explored cultural variations in rule following (cultural tightness or looseness^17^) as a possible contributor to the increased paranoia that we observed. State tightness may originate in response to threats like natural disasters, disease, territorial, and ideological conflict^17^. Tighter states typically evince more coordinated threat responses^17^. They have also experienced greater mortality from pneumonia and influenza throughout their history^17^. However, paranoia was highest in tight states with a mandate, with lower mask adherence during reopening. It may be that societies that adhere rigidly to rules are less able to adapt to unpredictable change. Alternatively, these societies may prioritize protection from ideological and economic threats over a public health crisis, or perhaps view the disease burden as less threatening.

Our analyses suggest that mandating mask-wearing may have caused paranoia to increase, altering participants’ expected volatility in the tasks (***μ***_3_^0^). Follow-up analyses suggested that in culturally tighter states with a mask mandate, those rules were being followed less (fewer people were wearing masks), inducing greater paranoia. Such violation of social norms engenders prediction errors^23^ which have been implicated in paranoia in laboratory studies^4, 24–26^.

### Public health implications

In economic games, compliance with social norms is often ensured through punishment^27, 28^. We note that during reopening, many states that mandated mask wearing were not enforcing it by punishing transgressors^29, 30^. Perhaps such punishments would increase compliance, with the added benefit of less norm violation and lower paranoia. However, given that paranoid individuals might be afraid of the consequences of their non-compliance, sanctions might backfire, resulting in vengeful acts^31^. Monetary or social incentives might increase compliance^32^, for example by promoting mask wearing as establishing a positive social image^33^, or providing compensatory moral praise^34^. Alternatively, tempering social expectations (by lowering priors on social reinforcement and compliance, ***μ***_2_^0^) such that norm violation is less salient, may mollify paranoia. This has been observed among the Berber people in the Atlas Mountains who trust less, and yet sustain cooperation^35^.

### Personal versus collective choices

Our findings are complex. Indeed, there is a seeming contradiction. On one hand, a more vigorous lockdown was associated with fewer sabotage beliefs. On the other hand, a more stringent mask wearing policy was associated with higher paranoia. How can strong rules have opposing effects on paranoia?

Perhaps a more vigorous lockdown provided fewer opportunities to misinterpret social interactions, whereas reopening provided more opportunities to encounter others and thence for paranoia. Abiding by lockdown is a personal choice whose effectiveness depends on ones’ own choice (to stay home and avoid others). Choosing to wear a mask also offers personal protection. However, mask wearing also protects others from the wearer; it is something one does for others.

Thus, mask-wearing is a collective action problem, wherein most people are *conditional cooperators*; generally willing to act in the collective interest as long as they perceive sufficient reciprocation by others^36^. Perceiving others refusing to follow the rules and failing to proffer reciprocal protection appears to have contributed to the increase in paranoia we observed. Indeed, paranoia, a belief in others’ nefarious intentions, also correlated with reluctance to wear a mask, and with endorsement of vaccine conspiracy theories. Finally, people who do not want to abide by the mask-wearing rules might be paranoid about being caught violating those rules. Lockdown may have offered fewer opportunities to be caught breaking the rules and therefore less paranoia.

### Non-social versus social mechanisms

It would be absurd to suggest that paranoia, by definition a social concern, is not undergirded by inferences about social features. Indeed, our data suggest that paranoia increases greatly when social rules are broken, particularly in cultures where rule-following is valued. However, we do not believe this is license to conclude that domain-specific coalitional mechanisms underwrite paranoia as some have argued^3^. Rather, our data show that both social and non-social inferences under uncertainty (particularly prior beliefs about volatility) are similarly related to paranoia. Further, they are similarly altered by real-world volatility, rules and rule breaking. We suggest that social inferences are instantiated by domain-general mechanisms^5, 37^. No doubt social inferences are important, difficult, and ill posed, but our data imply that they tax general inferential mechanisms rather than their own dedicated processes.

### Caveats

Whilst we independently (and multiply) replicated the associations between concerns about interventions that might mitigate the pandemic, paranoia and task behavior, and we show that our results are not driven by other real-world events, or issues with our sampling, there remain a number of important caveats to our conclusions. We did not run a within-subject study through the pandemic periods, however DiD analyses require longitudinal but not necessarily within-subjects or panel data^14^. Our DiD analysis does leverage some tentative causal claims, despite being based on between-subjects data^14^. The DiD analysis was warranted given that mask-mandate versus mask recommended states were matched at baseline in terms of COVID cases and deaths, as well as participant demographics. There are two key baseline differences between mandate and recommended states; recommended states were culturally tighter and more rural (t=−7.94, p=4.6E-11). Urbanicity is a key contributor to paranoia^38, 39^, though of course both cultural tightness and urbanicity did not change during the course of our study. Tightness did interact with mandate and adherence to mask wearing policy (Figure 5). The baseline difference in tightness would have worked against the effects we observed, not in their favor. Indeed, our multiple regression analysis found no evidence for an effect of tightness on paranoia in states without a mask-mandate (Figure 5). Critically, we do not know if any participant, or anyone close to them, was infected by COVID-19, so our work cannot speak to the more direct effects of infection. Finally, our work is based entirely in the USA. In future work we will expand our scope internationally. Cultural features^40^ and pandemic responses vary across nations. This variance should be fertile grounds in which to replicate and extend our findings.

### Conclusions

We highlight the impact that societal volatility and local cultural and policy differences have on individual cognition. This may have contributed to past failures to replicate in psychological research. If replication attempts were conducted under different economic, political or social conditions (bull versus bear markets, for example), then they may yield different results, not because of inadequacy of the theory or experiment but because the participants’ behavior was being modulated by heretofore under-appreciated stable and volatile local cultural features.

Per predictive processing theories^4^, paranoia increased with increases in real-world volatility, as did task-based priors and updating. Those effects were moderated by government responses. On one hand, proactive leadership mollified paranoia during lockdown, by tempering expectations of positive outcomes and volatility. On the other hand, mask mandates enhanced paranoia during reopening by imposing a rule that was often violated. These findings may help guide responses to future crises.

## Methods

All experiments were conducted at the Connecticut Mental Health Center in strict accordance with Yale University’s Human Investigation Committee. Informed consent was provided by all research participants.

### Experiment.

A total of 1,010 participants were recruited online via CloudResearch – an online research platform that integrates with MTurk while providing additional security for easy recruitment^21^. Two important studies were conducted to investigate paranoia and belief updating: pandemic study and replication study. ***Pandemic study***. A total of 605 participants were collected, divided into 202 pre-lockdown participants, 231 lockdown participants, and 172 reopening participants. Of the 202, we included the 72 (16 high paranoia) participants who completed the non-social task (described in a prior publication^5^). Those participants paranoia was self-rated with the SCID-II paranoid trait questions, which are strongly overlapping and correlated with the Green et al scale^5^. See Table 1 for further information. We recruited 130 (20 high paranoia) participants who completed the social task. Similarly, of the 231 (see Table 2 for details), we recruited 119 (27 high paranoia) and 112 (23 high paranoia) participants who completed the non-social and social tasks, respectively. Lastly, of the 172, we recruited 93 (35 high paranoia) and 79 (35 high paranoia) participants who completed the non-social and social tasks, respectively (See Table 3 for details). In addition to CloudResearch’s safeguard from bot submissions, we implemented the same study advertisement, submission review, approval and bonusing as described in our previous study^5^. We excluded a total of 163 submissions – 18 from pre-lockdown (social only), 34 from lockdown (non-social and social), and 111 from reopening (non-social and social). Of the 18, 17 were excluded based on incomplete/nonsensical free-response submissions and 1 for insufficient questionnaire completion. Of the 34, 29 were excluded based on incomplete/nonsensical free-response submissions and 5 for insufficient questionnaire completion. Of the 111, all were excluded based on incomplete/nonsensical free-response submissions. Submissions with grossly incorrect completion codes were rejected without further review. ***Replication study***. We collected a total of 405 participants of which 314 were low paranoid individuals and 91 were high paranoid individuals. Similar exclusion and inclusion criteria were applied for recruitment; most notably, we leveraged Cloud Research’s newly added *Data Quality* feature which only allows vetted high-quality participants – individuals who have passed their screening measures – into our study. This systematically cleaned all poor participants from our sample pool.

### Behavioral tasks.

Participants completed a 3-option probabilistic reversal-learning task with a non-social (card deck) or social (partner) domain frame. ***Non-social***: Three decks of cards were presented for 160 trials, divided evenly into 4 blocks. Each deck contained different amounts of winning (+100) and losing (−50) cards. Participants were instructed to find the best deck and earn as many points as possible. It was also noted that the best deck could change^11^. ***Social***: Three avatars were presented for 160 trials, divided evenly into 4 blocks. Participants were advised to imagine themselves as students at a university working with classmates to complete a group project, where some classmates were known to be unreliable – showing up late, failing to complete their work, getting distracted for personal reasons – or deliberately sabotage their work. Each avatar either represented a helpful (+100) or hurtful (−50) partner. We instructed participants to select an avatar (or partner) to work with to gain as many points towards their group project. Like the non-social, they were instructed that the best partner could change. For both tasks, the contingencies began as 90% reward, 50% reward, and 10% reward with the allocation across deck/partner switching after 9 out of 10 consecutive rewards. At the end of the second block, unbeknownst to the participants, the underlying contingencies transition to 80% reward, 40% reward, and 20% reward – making it more difficult to discern whether a loss of points was due to normal variations (probabilistic noise) or whether the best option has changed.

### Questionnaires.

Following task completion, questionnaires were administered via Qualtrics, we queried demographic information (age, gender, educational attainment, ethnicity, and race) and mental health questions (past or present diagnosis, medication use, *Structured Clinical Interview for DSM-IV Axis II Personality Disorders* (SCID-II)^8^, Beck’s Anxiety Inventory (BAI)^41^, Beck’s Depression Inventory (BDI)^42^, the Dimensional Obsessive-Compulsive Scale (DOCS)^19^, and critically, the revised Green et al., Paranoid Thoughts Scale (R-GPTS)^6^ – dividing clinically from non-clinically paranoid individuals based on the ROC-recommended cut-off score of 11 – and an additional item pertaining to their beliefs about the social task (‘Did any of the partners deliberately sabotage you?’) – on a Likert scale from ‘Definitely not’ to ‘Definitely yes’.

For the replication study, we adopted a survey^43^ that investigated beliefs on mask usage of individual US consumers and a survey^44^ of COVID-19. The 9-item mask questionnaire was used for our study to compute mask attitude (values < 0 indicate attitude against mask-wearing and values > 0 indicate attitude in favor of mask-wearing) for identifying group differences in paranoia. To compute an individual’s coronavirus vaccine conspiracy belief, we aggregated five vaccine-related questions from the 48-item coronavirus conspiracy questionnaire:

1. The coronavirus vaccine will contain microchips to control the people.
2. Coronavirus was created to force everyone to get vaccinated.
3. The vaccine will be used to carry out mass sterilization.
4. The coronavirus is bait to scare the whole globe into accepting a vaccine that will introduce the ‘real’ deadly virus.
5. The WHO already has a vaccine and are withholding it.

We adopted a 7-point scale: strongly disagree (1), disagree (2), somewhat disagree (3), neutral (4), somewhat agree (5), agree (6) and strongly agree (7). A higher score indicates greater endorsement of a question.

### Additional features.

Along with the task and questionnaire data, we examined state-level unemployment rate^45^, confirmed COVID-19 cases^46^, and mask usage^18^ in the USA. **Unemployment**. The *Carsey School of Public Policy* reported unemployment rates for the months of February, April, May and June in 2020. We utilized the rates in April and June as our markers for measuring the difference in unemployment between the pre-pandemic period and pandemic period, respectively. **Confirmed cases**. The *New York Times* published cumulative counts of coronavirus cases since January. We computed the mean cases per pandemic period with the following normalization approach:

(1) $$z_{i}=\frac{x_{i}-\text{min}(x)}{\text{max}(x)-\text{min}(x)}$$

where x represents our mean cases and *z*_*i*_ represents our *i*^*th*^ normalized data. **Mask wearing**. Similarly, at the request of the *New York Times*, *Dynata* – a research firm – conducted interviews on mask use across the USA and obtained a sample of 250,000 survey respondents between July 2 and July 14^18^. Each participant was asked: *How often do you wear a mask in public when you expect to be within six feet of another person?* The answer choices to the question included *Never, Rarely, Sometimes, Frequently*, and *Always*.

### Mask Policies.

According to the Philadelphia Inquirer: https://fusion.inquirer.com/health/coronavirus/covid-19-coronavirus-face-masks-infection-rates-20200624.html, 11 states mandated mask-wearing in public: CA, NM, MI, IL, NY, MA, RI, MD, VA, DE, and ME at the time of our reopening data collection. The other states from which we recruited participants recommended mask wearing in public.

### Protests.

We accessed the publicly available data from the armed conflict location and event data project (ACLED, https://acleddata.com/special-projects/us-crisis-monitor/), which has been recording the location, participation, and motivation of protests in the US since the week of George Floyd’s killing in May.

### Behavioral analysis.

We analysed tendencies to choose alternative decks after positive feedback (win-switch) and select the same deck after negative feedback (lose-stay). Win-switch rates were calculated as the number of trials in which the participant switched after positive feedback divided by the number of trials in which they received positive feedback. Lose-stay rates were calculated as number of trials in which a participant persisted after negative feedback divided by total negative feedback trials.

We also defined a proactivity metric (or score) to measure how adequately or inadequately a state reacted to COVID-19^47^. This score was calculated based on two features:

***Introduced***_***score***_ : number of days from baseline to introduce the stay-at-home order (i.e., baseline date – introduced date).
***Expiration***_***score***_ : number of days before the order was lifted (i.e., expiration date – introduced date).

where baseline date is defined as the date at which the first stay-at-home order was implemented. California was the first to enforce the order on March 19^th^, 2020 (i.e., baseline date = 0). States where stay-at-home orders were not implemented had ‘N/A’ values and were set to 0 in our calculation. Moreover, states that had an indefinite time frame for the orders were set to 100 in our calculation (i.e., expiration date = 100).

To compute the proactivity score, we perform the following sum:

(3) $$\text{Proactivit}y_{\text{score}\;}=\text{Introduce}d_{\text{score}}+\text{Expiratio}n_{\text{score}}$$

This metric – ranging from 0 (inadequate) to 100 (adequate) – offers a reasonable approach for measuring proactive state interventions in response to the pandemic.

### Causal inference.

To measure attribution of mask-wearing policy on paranoia, we adopt a differences-in-differences (DiD) approach. The DiD model we used to assess the causal effect of mask-wearing policy on paranoia from lockdown to reopening is represented below by the following equation:

(4) $$P_{i}=\alpha +\beta T_{i}+\gamma t_{i}+\delta \left(T_{i}*t_{i}\right)+\epsilon_{i}$$

where α is the constant term, β is the treatment group effect, γ is the time period common to both the control and treatment groups, and δ is the true causal effect. The control and treatment groups, in our case, represent states that recommend and require mask-wearing, respectively. The interaction term between the time covariate and mask-wearing represents our DiD estimate.

### Multiple regression analysis.

We conducted a multiple linear regression analysis, attempting to predict paranoia based on three continuous state variables – number of COVID-19 cases, cultural tightness and looseness (CTL) index, and mask-wearing belief – and one categorical state variable – mask policy. We fit a 15-predictor paranoia model on our N=172 individuals collected during reopening and proceeded to implement backward stepwise regression to find the model that best explains our data. Below we illustrate the full 15-predictor model and the resulting reduced 11-predictor model:

**Full model**:

$$\hat{y}=\beta_{0}+\beta_{1}*{\text{X}}_{\text{CASES}}+\beta_{2}*{\text{X}}_{\text{POLICY}}+\beta_{3}*{\text{X}}_{\text{CTL}}+\beta_{4}*{\text{X}}_{\text{MASK}}+\beta_{5}*{\text{X}}_{\text{CASES}*\text{POLICY}}+\beta_{6}*{\text{X}}_{\text{CASES}*\text{CTL}}\;+\beta_{7}*{\text{X}}_{\text{POLICY}*\text{CLT}}+\beta_{8}*{\text{X}}_{\text{CASES}*\text{MASK}}+\beta_{9}*{\text{X}}_{\text{CTL}*\text{MASK}}+\beta_{10}*{\text{X}}_{\text{CTL}*\text{MASK}}+\beta_{11}\;*{\text{X}}_{\text{CASES}*\text{POLICY}*\text{CTL}}+\beta_{12}*{\text{X}}_{\text{CASES}*\text{POLICY}*\text{MASK}}+\beta_{13}*{\text{X}}_{\text{CASES}*\text{CTL}*\text{MASK}}+\beta_{14}\;*{\text{X}}_{\text{POLICY}*\text{CTL}*\text{MASK}}+\beta_{15}*X_{\text{CASES}*\text{POLICY}*\text{CTL}*\text{MASK}}+\varepsilon$$

**Reduced model**:

$$\hat{y}=\beta_{0}+\beta_{1}*{\text{X}}_{\text{CASES}}+\beta_{2}*{\text{X}}_{\text{POLICY}}+\beta_{3}*{\text{X}}_{\text{CTL}}+\beta_{4}*{\text{X}}_{\text{MASK}}+\beta_{5}*{\text{X}}_{\text{CASES}*\text{POLICY}}+\beta_{6}*{\text{X}}_{\text{CASES}*\text{CTL}}\;+\beta_{7}*{\text{X}}_{\text{POLICY}*\text{CTL}}+\beta_{8}*{\text{X}}_{\text{POLICY}*\text{MASK}}+\beta_{9}*{\text{X}}_{\text{CTL}*\text{MASK}}+\beta_{10}*{\text{X}}_{\text{CASES}*\text{POLICY}*\text{CTL}}+\beta_{11}\;*{\text{X}}_{\text{POLICY}*\text{CTL}*\text{MASK}}+\varepsilon$$

See Table 7.

### Computational modeling.

The Hierarchical Gaussian Filter (HGF) toolbox v5.3.1 is freely available for download in the TAPAS package at https://translationalneuromodeling.github.io/tapas^9, 10^. We installed and ran the package in MATLAB and Statistics Toolbox Release 2016a (MathWorks ®, Natick, MA). We estimated perceptual parameters individually for the first and second halves of the task (i.e., blocks 1 and 2). Each participant’s choices (i.e., deck 1, 2, or 3) and outcomes (win or loss) were entered as separate column vectors with rows corresponding to trials. Wins were encoded as ‘1’, losses as ‘0’, and choices as ‘1’, ‘2’, or ‘3’. We selected the autoregressive 3-level HGF multi-arm bandit configuration for our perceptual model and paired it with the softmax-mu03 decision model. Table 4 describes the model parameter estimates from each study period.

### Statistics.

Statistical analyses and effect size calculations were performed with an alpha of 0.05 and two-tailed p-values in IBM SPSS Statistics, Version 25 (IBM Corp., Armonk, NY) and in RStudio: Integrated Development Environment for R, Version 1.3.959.

Independent samples t-tests were conducted to compare questionnaire item responses between high and low paranoia groups. Distributions of demographic and mental health characteristics across paranoia groups were evaluated by Chi-Square Exact tests (two groups) or Monte Carlo tests (more than 2 groups). Correlations were computed with Pearson’s rho.

HGF parameter estimates and behavioral patterns (win-switch and lose-stay rates) were analyzed by repeated measures and split-plot ANOVAs (i.e., block designated as within-subject factor; pandemic, paranoia group, and social versus non-social condition as between subject factors). Model parameters were corrected for multiple comparisons using the Benjamini Hochberg^48^ method with a false discovery rate of 0.05 in analyses of variance across experiments. We performed ANCOVAs for model parameters using three sets of covariates: (1) demographics (age, gender, ethnicity, and race); (2) mental health factors (medication usage, diagnostic category, BAI score, and BDI score); (3) and metrics and correlates of global cognitive function (educational attainment, income, and cognitive reflection). Post-hoc tests were conducted as least significant difference (LSD)-corrected estimated marginal means. See Tables 5 and 6 for more details.

To conduct meta-analyses of effect replication across experiments, we fit random effects models in the R Metafor package^49^. Mean differences of low versus high paranoia groups were calculated for social and non-social pre-pandemic experiments.

## Extended Data

**Extended Data Table 1:** Subject characteristics by experimental condition during the pre-pandemic period.

	Pre-pandemic					
	Nonsocial			Social		
	Low paranoia (n=56)	High paranoia (n=16)	*P*, Statistic, df	Low paranoia (n=110)	High paranoia (n=20)	*P*, Statistic, df
**Demographics**						
Age (years)^a^	38.6 [11.7]	32.9 [7.0]	0.019, −2.4^b^*,42*	39.7 [11.5]	32.5 [7.0]	5.6E-4, −3.7^b^, 41
Gender			0.377, 0.78^d^, 1			0.023, 5.13^d^, 1
% Female	50.0	62.5	n/a	47.3	20.0	n/a
% Male	50.0	37.5	n/a	52.7	80.0	n/a
% Other or not specified	0.0	0.0	n/a	0.0	0.0	n/a
Ethnicity			0.732, 0.12^d^, 1			0.002, 9.9^d^, 1
% Hispanic, Latino, Spanish	8.9	6.2	n/a	2.7	20.0	n/a
% Not Hispanic, Latino, Spanish	91.1	93.8	n/a	97.3	80.0	n/a
% Not specified	0.0	0.0	n/a	0.0	0.0	n/a
Race			0.084, 9.7^d^, 5			0.135, 7.0^d^, 4
% White	85.7	75.0	n/a	80.0	65.0	n/a
% Black or African American	0.0	12.5	n/a	10.0	30.0	n/a
% Asian	3.6	6.2	n/a	3.6	5.0	n/a
% American Indian or Alaska Native	1.8	6.2	n/a	0.0	0.0	n/a
% Multiracial	3.6	0.0	n/a	5.5	0.0	n/a
% Other or not specified	5.4	0.0	n/a	0.9	0.0	n/a
**Cognitive Function**						
Education			0.500, 5.4^d^, 6			0.655, 3.3^d^, 5
% High school / equivalent	16.1	6.2	n/a	16.4	5.0	n/a
% Some college or university	17.9	25.0	n/a	17.3	20.0	n/a
% Associate’s degree	12.5	12.5	n/a	10.9	15.0	n/a
% Bachelor’s degree	35.7	56.2	n/a	42.7	55.0	n/a
% Master’s degree	14.3	0.0	n/a	11.8	5.0	n/a
% Doctoral or professional	1.8	0.0	n/a	0.0	0.0	n/a
% Postgraduate	1.8	0.0	n/a	0.9	0.0	n/a
% Not specified	0.0	0.0	n/a	0.0	0.0	n/a
Income			0.636, 3.4^d^, 5			0.494, 4.4^d^, 5
% Less than $20,000	17.9	37.5	n/a	11.8	0.0	n/a
% $20,000 to $34,999	33.9	31.3	n/a	25.5	20.0	n/a
% $35,000 to $49,999	12.5	6.3	n/a	17.3	20.0	n/a
% $50,000 to $74,999	21.4	33.3	n/a	23.6	35.0	n/a
% $75,000 to $99,999	8.9	6.2	n/a	11.8	20.0	n/a
%Over $100,000	3.6	6.2	n/a	7.3	5.0	n/a
%Not specified	1.8	0.0	n/a	2.7	0.0	n/a
Cognitive Reflection^a^	2.09 [1.16]	1.50 [1.15]	0.078, −1.8^c^, 70	2.05 [ 1.04]	1.4 [0.94]	0.01, −2.6^c^, 128
**Mental Health**						
Psychiatric diagnosis			0.022, 9.7^d^, 3			6.5E-4, 17.2^d^, 3
% No history of mental illness	71.4	43.8	n/a	62.7	40.0	n/a
% Schizophrenia spectrum	0.0	6.2	n/a	0.0	5.0	n/a
% Mood disorder	16.1	43.8	n/a	26.4	15.0	n/a
% Other, not specified	12.5	6.2	n/a	10.9	40.0	n/a
Psychotropic medication (%)	7.14	25.0	0.083, 6.7^d^, 3	9.1	15.0	0.075, 6.9^d^, 3
Beck’s Anxiety Inventory^a^	0.236 [0.292]	0.903 [0.793]	0.004, 3.3^b^, 16	0.355 [0.460]	0.926 [0.617]	6.4E-4, 3.9^b^, 23
Beck’s Depression Inventory^a^	0.248 [0.336]	1.031 [0.772]	0.001, 4.0^b^, 17	0.428 [0.522]	1.085 [0.621]	1.6E-4, 4.5^c^, 24
SCID Paranoid Personality^a^	0.097 [0.131]	0.725 [0.144]	2.2E-16, 16.5^c^, 70	n/a	n/a	n/a
Green et al. Paranoid Thoughts Scale, revised^a,e^	n/a	n/a	n/a	0.194 [0.291]	2.038 [0.596]	9.5E-12, 13.5^b^,21

a, mean [standard deviation]

b, t-statistic, degrees of freedom (equal variances not assumed)

c, t-statistic, degrees of freedom, equal variances assumed

d, Pearson Chi-square, degrees of freedom

e, Normalized GPTS score

**Extended Data Table 2:** Subject characteristics by experimental condition during the lockdown period.

	Lockdown					
	Nonsocial			Social		
	Low paranoia (n=92)	High paranoia (n=27)	*P*, Statistic, df	Low paranoia (n=89)	High paranoia (n=23)	*P*, Statistic, df
**Demographics**						
Age (years)^a^	38.8 [11.9]	37.4 [9.2]	0.530, −0.6^b^, 54	37.2 [10.2]	37.0 [11.7]	0.933, −0.08^b^, 31
Gender			0.665, 0.82^d^, 2			0.492, 1.4^d^, 2
% Female	31.5	37.0	n/a	43.8	39.1	n/a
% Male	66.3	63.0	n/a	51.7	60.9	n/a
% Other or not specified	2.2	0.0	n/a	4.5	0.0	n/a
Ethnicity			0.703, 0.15^d^, 1			0.438, 0.60^d^, 1
% Hispanic, Latino, Spanish	8.7	11.1	n/a	7.9	13.0	n/a
% Not Hispanic, Latino, Spanish	91.3	88.9	n/a	92.1	87.0	n/a
%Not specified	0.0	0.0	n/a	0.0	0.0	n/a
Race			0.639, 3.4^d^, 5			0.593, 2.8^d^, 4
% White	83.7	81.5	n/a	76.4	82.6	n/a
% Black or African American	6.5	7.4	n/a	15.7	13.0	n/a
% Asian	2.2	7.4	n/a	5.6	0.0	n/a
% American Indian or Alaska Native	1.1	0.0	n/a	0.0	0.0	n/a
% Multiracial	2.2	3.7	n/a	1.1	0.0	n/a
% Other or not specified	4.3	0.0	n/a	1.1	4.3	n/a
**Cognitive Function**						
Education			0.256, 7.76^d^, 6			0.864, 2.5^d^, 6
% High school / equivalent	15.2	14.8	n/a	6.7	4.3	n/a
% Some college or university	19.6	11.1	n/a	21.3	13.0	n/a
% Associate’s degree	13.0	14.8	n/a	16.9	17.4	n/a
% Bachelor’s degree	39.1	51.9	n/a	42.7	52.2	n/a
% Master’s degree	9.8	0.0	n/a	10.1	8.7	n/a
% Doctoral or professional	3.3	3.7	n/a	1.1	0.0	n/a
% Postgraduate	0.0	3.7	n/a	1.1	4.3	n/a
% Not specified	0.0	0.0	n/a	0.0	0.0	n/a
Income			0.421, 4.96^d^, 5			0.099, 10.7^d^, 6
% Less than $20,000	17.4	33.3	n/a	13.5	8.7	n/a
% $20,000 to $34,999	23.9	11.1	n/a	27.0	26.1	n/a
% $35,000 to $49,999	17.4	22.2	n/a	20.2	8.7	n/a
% $50,000 to $74,999	21.7	18.5	n/a	27.0	34.8	n/a
% $75,000 to $99,999	10.9	11.1	n/a	4.5	21.7	n/a
%Over $100,000	7.6	3.7	n/a	6.7	0.0	n/a
%Not specified	1.1	0.0	n/a	1.1	0.0	n/a
Cognitive Reflection^a^	1.98 [1.10]	1.89 [1.12]	0.712, −0.37^c^, 117	1.75 [1.19]	1.96 [1.19]	0.466, 0.73^c^, 110
**Mental Health**						
Psychiatric diagnosis			0.062, 7.32^d^, 3			0.009, 9.42^d^, 2
% No history of mental illness	55.4	77.8	n/a	59.6	52.2	n/a
% Schizophrenia spectrum	1.1	0.0	n/a	0.0	0.0	n/a
% Mood disorder	23.9	22.2	n/a	23.6	4.3	n/a
% Other, not specified	19.6	0.0	n/a	16.9	43.5	n/a
Psychotropic medication (%)	10.9	11.1	0.123, 5.78^d^, 3	6.7	4.3	0.551, 2.11^d^, 3
Beck’s Anxiety Inventory^a^	0.421 [0.553]	0.337 [0.589]	0.512, −0.66^b^, 40	0.627 [0.691]	0.412 [0.606]	0.148, −1.48^b^, 38
Beck’s Depression Inventory^a^	0.491 [0.609]	0.372 [0.602]	0.374, −0.90^b^, 43	0.701 [0.747]	0.340 [0.429]	0.004, −3.03^b^, 61
SCID Paranoid Personality^a^	n/a	n/a	n/a	n/a	n/a	n/a
Green et al. Paranoid Thoughts Scale, revised^a,e^	0.177 [0.305]	2.05 [0.536]	2.2E-16, 17.3^b^, 31	0.202 [0.295]	2.10 [0.701]	3.9E-12, 12.7^b^, 24

a, mean [standard deviation]

b, t-statistic, degrees of freedom (equal variances not assumed)

c, t-statistic, degrees of freedom, equal variances assumed

d, Pearson Chi-square, degrees of freedom

e, Normalized GPTS score

**Extended Data Table 3:** Subject characteristics by experimental condition during the reopening period.

	Reopening					
	Nonsocial			Social		
	Low paranoia (n=58)	High paranoia (n=35)	*P*, Statistic, df	Low paranoia (n=44)	High paranoia (n=35)	*P*, Statistic, df
**Demographics**						
Age (years)^a^	39.7 [13.1]	33.5 [9.6]	0.011, −2.6^c^, 83	34.7 [7.9]	33.7 [8.2]	0.569, −0.57^c^, 66
Gender			0.400, 0.71^d^, 1			0.085, 4.9^d^, 2
% Female	39.7	48.6	n/a	47.7	25.7	n/a
% Male	60.3	51.4	n/a	52.3	71.4	n/a
% Other or not specified	0.0	0.0	n/a	0.0	2.9	n/a
Ethnicity			0.113, 2.5^d^, 1			0.507, 1.36^d^, 2
% Hispanic, Latino, Spanish	8.6	20.0	n/a	13.6	17.1	n/a
% Not Hispanic, Latino, Spanish	91.4	80.0	n/a	84.1	82.9	n/a
%Not specified	0.0	0.0	n/a	2.3	0.0	n/a
Race			0.232, 6.9^d^, 5			0.662, 3.2^d^, 5
% White	75.9	85.7	n/a	77.3	82.9	n/a
% Black or African American	6.9	8.6	n/a	11.4	8.6	n/a
% Asian	6.9	0.0	n/a	2.3	5.7	n/a
% American Indian or Alaska Native	1.7	5.7	n/a	4.5	0.0	n/a
% Multiracial	5.2	0.0	n/a	2.3	2.9	n/a
% Other or not specified	3.4	0.0	n/a	2.3	0.0	n/a
**Cognitive Function**						
Education			0.065, 11.9^d^, 6			0.061, 10.6^d^, 5
% High school / equivalent	12.1	8.6	n/a	11.4	11.4	n/a
% Some college or university	20.7	14.3	n/a	27.3	11.4	n/a
% Associate’s degree	17.2	2.9	n/a	11.4	0.0	n/a
% Bachelor’s degree	32.8	65.7	n/a	40.9	51.4	n/a
% Master’s degree	12.1	8.6	n/a	9.1	22.9	n/a
% Doctoral or professional	3.4	0.0	n/a	0.0	2.9	n/a
% Postgraduate	1.7	0.0	n/a	0.0	0.0	n/a
% Not specified	0.0	0.0	n/a	0.0	0.0	n/a
Income			0.799, 2.4^d^, 5			0.171, 7.7^d^, 5
% Less than $20,000	17.2	11.4	n/a	15.9	2.9	n/a
% $20,000 to $34,999	20.7	14.3	n/a	20.5	20.0	n/a
% $35,000 to $49,999	20.7	31.4	n/a	25	20.0	n/a
% $50,000 to $74,999	25.9	28.6	n/a	20.5	37.1	n/a
% $75,000 to $99,999	10.3	11.4	n/a	4.5	14.3	n/a
%Over $100,000	5.2	2.9	n/a	9.1	5.7	n/a
%Not specified	0.0	0.0	n/a	4.5	0.0	n/a
Cognitive Reflection^a^	1.90 [1.04]	0.77 [0.97]	1.3E-6, −5.2^c^, 91	1.86 [1.09]	1.09 [1.09]	0.002, −3.1^c^, 77
**Mental Health**						
Psychiatric diagnosis			0.028, 7.1^d^, 2			0.415, 1.8^d^, 2
% No history of mental illness	56.9	28.6	n/a	36.4	25.7	n/a
% Schizophrenia spectrum	0.0	0.0	n/a	0.0	0.0	n/a
% Mood disorder	19	34.3	n/a	31.8	28.6	n/a
% Other, not specified	24.1	37.1	n/a	31.8	45.7	n/a
Psychotropic medication (%)	8.6	2.9	0.041, 8.3^d^, 3	11.4	17.1	0.322, 3.5^d^, 3
Beck’s Anxiety Inventory^a^	0.325 [0.407]	1.21 [0.782]	1.5E-7, 6.2^b^, 45	0.441 [0.464]	0.826 [0.703]	0.007, 2.8^b^, 56
Beck’s Depression Inventory^a^	0.326 [0.407]	1.19 [0.713]	3.3E-8, 6.6^b^, 48	0.496 [0.601]	0.850 [0.609]	0.012, 2.6^b^, 73
SCID Paranoid Personality^a^	n/a	n/a	n/a	n/a	n/a	n/a
Green et al. Paranoid Thoughts Scale, revised^a,e^	0.248 [0.307]	2.187 [0.473]	2.2E-16, 21.7^b^, 51	0.196 [0.276]	2.189 [0.532]	2.2E-16, 20^b^, 48

a, mean [standard deviation]

b, t-statistic, degrees of freedom (equal variances not assumed)

c, t-statistic, degrees of freedom, equal variances assumed

d, Pearson Chi-square, degrees of freedom

e, Normalized GPTS score

**Extended Data Table 4:** Behavior and model parameters by paranoia group and pandemic period.

	Low Paranoia		High Paranoia	
	Block 1 Mean (SD)	Block 2 Mean (SD)	Block 1 Mean (SD)	Block 2 Mean (SD)
**Pre-pandemic**^a^				
Win-switch rate	0.059 (0.115)	0.043 (0.095)	0.185 (0.229)	0.147 (0.190)
Lose-stay rate	0.275 (0.232)	0.290 (0.222)	0.312 (0.222)	0.325 (0.203)
***μ***_3_^0^	−0.223 (1.290)	−1.500 (1.503)	0.410 (0.677)	−0.862 (1.715)
***ω***_3_	−0.287 (1.085)	−1.046 (0.863)	−0.698 (1.257)	−1.287 (0.819)
***κ***_2_^0^	−0.151 (0.269)	−0.314 (0.370)	−0.093 (0.134)	−0.295 (0.444)
***ω***_2_	1.190 (1.366)	1.081 (1.292)	0.211 (1.499)	0.406 (1.604)
***κ***	0.494 (0.069)	0.467 (0.071)	0.553 (0.075)	0.514 (0.086)
**Lockdown**^b^				
Win-switch rate	0.132 (0.218)	0.090 (0.180)	0.130 (0.264)	0.094 (0.214)
Lose-stay rate	0.245 (0.201)	0.267 (0.215)	0.274 (0.250)	0.276 (0.239)
***μ***_3_^0^	−0.039 (1.225)	−1.301 (1.648)	−0.206 (1.318)	−1.369 (1.786)
***ω***_3_	−0.428 (1.145)	−0.928 (0.959)	−0.570 (1.191)	−1.153 (0.811)
***μ***_2_^0^	−0.133 (0.218)	−0.270 (0.391)	−0.178 (0.267)	−0.285 (0.474)
***ω***_2_	0.933 (1.524)	0.791 (1.433)	0.758 (1.570)	0.754 (1.458)
***κ***	0.510 (0.080)	0.482 (0.078)	0.511 (0.078)	0.481 (0.090)
**Reopening**^c^				
Win-switch rate	0.061 (0.131)	0.042 (0.089)	0.239 (0.276)	0.176 (0.243)
Lose-stay rate	0.285 (0.233)	0.300 (0.209)	0.152 (0.172)	0.183 (0.203)
***μ***_3_^0^	−0.333 (1.248)	−1.809 (1.494)	0.607 (0.581)	−0.191 (1.295)
***ω***_3_	−0.212 (1.112)	−0.918 (0.870)	−0.866 (1.061)	−1.293 (0.883)
***μ***_2_^0^	−0.180 (0.279)	−0.366 (0.429)	−0.020 (0.086)	−0.080 (0.183)
***ω***_2_	1.281 (1.210)	1.055 (1.070)	0.527 (1.778)	0.694 (1.816)
***κ***	0.450 (0.073)	0.462 (0.064)	0.521 (0.087)	0.508 (0.094)

a, n=166 low paranoia, 36 high paranoia

b, n=181 low paranoia, 50 high paranoia

c, n=102 low paranoia, 70 high paranoia

**Extended Data Table 5:** ANOVAs across experiments.

	Split-plot ANOVA^a^						
	WSR^c^	LSR^d^	*μ*_3_^0^	*ω*_3_	*μ*_2_^0^	*ω*_2_	*κ*
Effect	*P* (F)	*P* (F)	*P* (F)	*P* (F)	*P* (F)	*P* (F)	*P* (F)
**Within-subject**							
block	1.19E-7^f,g,h^ (28.729)	0.024^f,g,h^ (5.141)	7.06E-92^e^ (598.165)	1.92E-21^e^ (97.778)	8.71E-19^e^ (83.816)	0.675 (0.175)	3.53E-16^f^ (70.413)
block*version	0.579 (0.308)	0.592 (0.287)	0.340 (0.911)	0.597 (0.280)	0.300 (1.076)	0.724 (0.125)	0.456 (0.556)
block*pandemic	0.589 (0.530)	0.760 (0.275)	0.533 (0.629)	0.643 (0.441)	0.284 (1.263)	0.723 (0.324)	0.615 (0.486)
block*paranoia	0.141 (2.178)	0.690 (0.159)	0.007^h,m^ (7.237)	0.251 (1.321)	0.220 (1.507)	0.02^g,m^ (5.446)	0.528 (0.400)
block*version*pandemic	0.586 (0.535)	0.948 (0.054)	0.246 (1.408)	0.820 (0.198)	0.996 (0.004)	0.583 (0.54)	0.859 (0.152)
block*version*paranoia	0.885 (0.021)	0.518 (0.418)	0.889 (0.02)	0.400 (0.709)	0.876 (0.024)	0.883 (0.022)	0.574 (0.317)
block*pandemic*paranoia	0.260 (1.350)	0.591 (0.526)	0.009^e,o^ (4.811)	0.348 (1.058)	0.079 (2.546)	0.579 (0.548)	0.104 (2.276)
block*version*pandemic*paranoia	0.624 (0.472)	0.187 (1.683)	0.993 (0.007)	0.419 (0.871)	0.853 (0.159)	0.463 (0.771)	0.799 (0.225)
**Between-subject**							
version	0.450 (0.572)	0.103 (2.66)	0.732 (0.117)	0.403 (0.700)	0.688 (0.162)	0.491 (0.476)	0.381 (0.768)
pandemic	0.349 (1.054)	0.005^f,g^ (5.419)	0.102 (2.291)	0.816 (0.203)	0.110 (2.220)	0.607 (0.500)	0.474 (0.748)
paranoia	4.3E-08^e^ (30.81)	0.268 (1.228)	1.2E-06^e,l^ (24.02)	1.3E-05^h,l^ (19.31)	0.006^e,l^ (7.501)	7.4E-05^e,l^ (15.93)	9.3E-06^e,l^ (19.99)
version*pandemic	0.189 (1.669)	0.258 (1.357)	0.595 (0.520)	0.827 (0.190)	0.333 (1.103)	0.958 (0.043)	0.902 (0.103)
version*paranoia	0.670 (0.182)	0.625 (0.239)	0.120 (2.429)	0.753 (0.099)	0.238 (1.394)	0.935 (0.007)	0.657 (0.197)
pandemic*paranoia	0.0001^e^ (9.08)	0.002^e^ (6.51)	6.9E-06^e,n^ (12.12)	0.152 (1.890)	0.0001^e,n^ (8.996)	0.058 (2.858)	0.003^e,n^ (5.766)
version*pandemic*paranoia	0.522 (0.652)	0.085 (2.474)	0.892 (0.114)	0.261 (1.347)	0.365 (1.011)	0.572 (0.559)	0.277 (1.288)

a across all conditions (pre-pandemic, lockdown and reopening; social and nonsocial versions). n=156 high paranoia, 449 low paranoia; df=1, error=593.

b data align-rank-transformed for non-parametric repeated measures ANOVA. df=1, error=593.

c Win-switch rate.

d Lose-stay rate.

e Survives ANCOVAs for demographic variables, correlates of cognitive ability, and mental health factors.

f Does not survive ANCOVA for demographic variables (age, gender, ethnicity, race).

g Does not survive ANCOVA for correlates of cognitive ability (educational attainment, income, cognitive reflection score).

h Does not survive ANCOVA for mental health variables (psychotropic medication use, psychiatric diagnosis, BAI score, BDI score).

i Does not survive correction for multiple comparisons with false discovery rate=0.05 (familywise for model parameters, block*paranoia effects).

j Does not survive correction for multiple comparisons with false discovery rate=0.05 (familywise for model parameters, pandemic*paranoia effects).

k Does not survive correction for multiple comparisons with false discovery rate=0.05 (familywise for model parameters, block*pandemic*paranoia effects).

l Survives correction for multiple comparisons with false discovery rate=0.05 (familywise for model parameters, paranoia effects).

m Survives correction for multiple comparisons with false discovery rate=0.05 (familywise for model parameters, block*paranoia effects).

n Survives correction for multiple comparisons with false discovery rate=0.05 (familywise for model parameters, pandemic*paranoia effects).

o Survives correction for multiple comparisons with false discovery rate=0.05 (familywise for model parameters, block*pandemic*paranoia effects).

**Extended Data Table 6:** Estimated marginal means for paranoia by pandemic period interactions.

		High versus low paranoia		
Parameter	Period	MD_EMM_	SE_EMM_	P-value
Win-switch rate	Pre-pandemic	0.116	0.031	0.0002
	Lockdown	<0.001	0.027	0.987
	Reopening	0.153	0.026	5.87E-09
Lose-stay rate	Pre-pandemic	0.034	0.038	0.362
	Lockdown	0.019	0.032	0.566
	Reopening	−0.118	0.031	0.0002
***μ***_3_^0^, block 1	Pre-pandemic	0.693	0.219	0.002
	Lockdown	−0.19	0.188	0.314
	Reopening	0.934	0.183	4.42E-07
***μ***_2_^0^	Pre-pandemic	0.037	0.052	0.475
	Lockdown	−0.036	0.044	4.20E-01
	Reopening	0.219	0.043	4.76E-07
***κ***	Pre-pandemic	0.055	0.013	1.67E-05
	Lockdown	<0.001	0.011	0.985
	Reopening	0.934	0.183	4.42E-07

## Figures and Tables

**Table 7.** Regression Analysis for Paranoia during Reopening

Variable	Full model	Reduced model
**CASES**	−6.12e-05	−2.43e-06
**POLICY**	−1.63e+02	−4.99e+01
**CTL**	−6.72e-02	−4.20e-02
**MASK**	−3.16	−8.45e-01
CASES*POLICY	1.55e-03	−1.70e-05
CASES*CTL	8.62e-07	−9.68e-09
POLICY*CTL	3.73	1.32*
CASES*MASK	7.81e-05	-
POLICY*MASK	2.16e+02	7.07e+01*
CTL*MASK	8.69e-02	5.51e-02
CASES*POLICY*CTL	−3.33e-05	4.98e-07
CASES*POLICY*MASK	−2.00e-03	-
CASES*CTL*MASK	−1.14e-06	-
POLICY*CTL*MASK	−4.98	−1.87*
CASES*POLICY*CTL*MASK	4.33e-05	-
**Adjusted R**^**2**^	0.04	0.06

* p ≤ .05,

** p ≤ .01
